# Butyrate Enhances Antimicrobial Defence in Chicken Macrophages Through Reactive Oxygen Species Generation and Autophagy Activation

**DOI:** 10.3390/cells14211742

**Published:** 2025-11-06

**Authors:** James R. G. Adams, Faisal R. Anjum, Jai W. Mehat, Roberto M. La Ragione, Shahriar Behboudi

**Affiliations:** 1 Discipline of Veterinary Clinical Sciences, School of Veterinary Medicine, Faculty of Health and Medical Sciences, University of Surrey, Guildford GU2 7AL, UK; j.r.adams@surrey.ac.uk; 2 The Pirbright Institute, Pirbright, Woking GU24 0NE, UK; 3 Bristol Veterinary School, University of Bristol, Langford BS40 5DU, UK; faisal.anjum@bristol.ac.uk; 4 Discipline of Microbes, Infection and Immunity, School of Biosciences, Faculty of Health and Medical Sciences, University of Surrey, Guildford GU2 7XH, UK; jw.mehat@surrey.ac.uk (J.W.M.); r.laragione@surrey.ac.uk (R.M.L.R.); 5 Discipline of Comparative Biomedical Sciences, School of Veterinary Medicine, Faculty of Health and Medical Sciences, University of Surrey, Guildford GU2 7AL, UK

**Keywords:** priming, APEC, macrophages, sodium butyrate, chicken, autophagy, ROS

## Abstract

Sodium butyrate has been documented to support gut function and help control pathogens in the gastrointestinal tract. However, the precise mechanisms of dietary sodium butyrate’s control over enteric pathogens in chickens remain unclear. Our study demonstrated that priming chicken bone marrow-derived macrophages (BMDMs) or the HD11 cell line with 1 mM sodium butyrate significantly enhanced their antimicrobial capacity against key bacterial pathogens (*Escherichia coli*, *Salmonella* Typhimurium, *Pseudomonas aeruginosa*, and *Staphylococcus aureus*) in gentamicin protection assays (*p* < 0.05; ≥1 log reduction in CFU/mL). This in vitro enhancement was associated with increased production of reactive oxygen species (ROS), as detected by DCFH-DA assays, showing approximately a 30% increase in HD11 cells and a 12% increase in BMDMs. Butyrate priming was observed to result in autophagy activation, potentially through mTOR pathway inhibition, evidenced by changes in related gene expression using RT-qPCR assay and a 2.5-fold increase in GFP-LC3B accumulation. Supporting this, pharmacological inhibition of ROS using the ROS scavenger N-acetyl-L-cystine (NAC) or autophagy with chloroquine reduced the butyrate-enhanced bacterial clearance. Furthermore, the mTOR inhibitor rapamycin synergized with butyrate priming, whereas the mTOR activator L-leucine counteracted enhanced antimicrobial activity. These findings offer crucial insights for improving host defence against bacterial infections and developing novel therapeutic strategies in chickens.

## 1. Introduction

Macrophages are critical effector cells within the innate immune system, playing a central role in defending against bacterial infections by engulfing and eliminating invading pathogens [1]. Furthermore, macrophages play a key role in gut homeostasis, removing dead cells and helping to maintain the epithelial layer [2]. These functions have been observed to be enhanced following immunomodulation, such as the induction of priming with immunostimulatory compounds, which results in a change in the functional immune status of target cells, which does not return to basal levels before the secondary stimulation or infection [3]. Toll-like receptor (TLR) ligands, such as ODN-CpG [4], and beta-glucan [5,6], have been demonstrated to modulate macrophage function, resulting in improved antimicrobial activity. However, these molecules have been associated with the induction of pro-inflammatory responses and immunopathologies [7], which may lead to inflammation that can hinder growth within chickens and other livestock [8]. Therefore, identifying molecules that selectively enhance the function of innate immune cells, particularly macrophages, without inducing detrimental inflammation is crucial. The short chain fatty acid (SCFA) sodium butyrate, found within the gastrointestinal tract as butyric acid, is a product of gut microbiota fermentation, predominantly produced by members of the Clostridium cluster of the Firmicutes phylum, and offers a promising avenue of investigation [9]. Incorporating butyrate into chicken diets at concentrations of 0.2% has demonstrated beneficial effects, including enhanced gut development, reduced inflammation, modulated gut microbiota, increased weight gain in broilers, improved feed digestion, and decreased *E. coli* and *Salmonella* colonisation [10,11,12]. While the exact mechanism by which butyrate controls enteric pathogens is still under investigation [13], it is purported to function mechanistically as a histone deacetylase inhibitor (HDACI) [14,15] and through activation of cell surface receptors. These include the G-protein-coupled receptors GPR109A, GPR41, and GPR43, whose activation triggers an anti-inflammatory response in colonic cells [16,17]. Butyrate’s established benefits for gut health have led to significant research into its use in animal production, particularly as an alternative to in-feed antibiotics. We hypothesise that one key mechanism by which butyrate controls enteric pathogens, including avian colibacillosis, is through immune priming, resulting in improved antibacterial functions of immune cells, especially chicken macrophages.

Avian colibacillosis is one of the leading causes of bird mortality and morbidity, presenting a global financial and animal health and welfare issue [18] and affecting every production system in all farmed species of poultry [19,20]. The *E. coli* associated with colibacillosis are referred to as avian pathogenic *E. coli* (APEC) and are considered a sub-pathotype of extra-intestinal pathogenic *E. coli* (ExPEC). Published reports on the geographical prevalence of APEC are scarce, but it is estimated that 30% of commercial flocks within the US are infected at any one time [21]. APEC has traditionally been treated with antibiotics, but this can drive the emergence of antimicrobial resistance (AMR) [22,23]. Vaccines against colibacillosis offer effective protection but remain costly and may have limited effectiveness against emerging novel APEC lineages [24,25]. Immune priming presents a promising approach to the management of APEC due to its capacity to enhance macrophage responses against a broad range of bacterial lineages and species [26]. Therefore, this study examines the priming effect of sodium butyrate on chicken macrophages and seeks to elucidate the mechanisms through which butyrate enhances macrophage function in controlling various pathotypes of APEC in vitro. Two models, the HD11 immortalised cell line and primary BMDMs, were employed to examine the effect of established and immature macrophages, respectively. Furthermore, the use of the HD11 immortalised cell line allows the use of plasmid transfection to investigate intracellular mechanisms. The results from this study may lead to improved management strategies against this highly prevalent bacterial pathogen in chickens.

## 2. Materials and Methods

### 2.1. Bacterial Isolates and Growth Conditions

The bacteria used within this study (Table 1) included APEC obtained from the Surrey Animal Pathogen (SAP) collection, isolates recovered from turkeys diagnosed with colibacillosis, avirulent Lab strain MG1655, *Salmonella* Typhimurium ST SL1344 WT, Methicillin-resistant *Staphylococcus aureus* (MRSA) NCTC 12493, and *Pseudomonas aeruginosa* NCTC 12903, also held within the SAP collection. Isolates were stored in Pro-Lab Diagnostics Microbank (Fisher, Basingstoke, UK) tubes at −80 °C. Prior to use, *E. coli* isolates were streaked onto MacConkey Agar No. 3 (Oxoid, Basingstoke, UK) while *S.* Typhimurium, MRSA, and *Pseudomonas aeruginosa* were streaked onto nutrient agar (Oxoid, Basingstoke, UK), before incubation at 37 °C, aerobically, for 16 h. For broth cultures, a single colony of each bacterial isolate was used to inoculate 10 mL of fresh LB broth in a sterile 50 mL Falcon tube before aerobic incubation at 37 °C, aerobically, with shaking at 225 RPM, for 16 h.

### 2.2. Preparation of Bacterial Inoculum for Tissue Culture Assays

A single colony of an isolate was used to inoculate 10 mL of fresh LB broth in a 50 mL sterile Falcon tube before aerobic incubation at 37 °C with shaking at 225 RPM for 16 h. Following incubation, 100 μL was removed and used to inoculate 9.9 mL LB in a 50 mL sterile centrifuge tube before culture for two hours at 37 °C, aerobically, with shaking at 225 RPM. Cultures were pelleted by centrifugation at 13,000× *g* for 15 min and media discarded before resuspension in phosphate-buffered saline (PBS) (7.4 pH, KH_2_PO_4_ 1 mM, NaCl 155 mM, Na_2_HPO_4_-7H_2_O, Gibco, ThermoFisher, Paisley, UK). Optical density was measured by spectrophotometer at 600 nm and the bacterial suspension was diluted in prewarmed media to achieve the desired multiplicity of infection (MOI). MOI was confirmed by calculation of CFU/mL using the Miles and Misra technique [27].

### 2.3. Growth and Maintenance of Chicken Macrophage-like HD11 Cells

The HD11 cell line [28] was maintained in Roswell Park Memorial Institute (RPMI) 1640 Medium with 5% foetal bovine serum (FBS), 5% chicken serum (CS), and 1% Penicillin/Streptomycin (ThermoFisher, Paisley, UK) at 37 °C + 5% CO_2_. When 80% confluency was reached, the cells were detached and used to seed 24-well plates at a concentration of 2 × 10^5^ cells/mL. Cells were incubated at 41 °C + 5% CO_2_ for 24 h.

### 2.4. Ethical Approval for Collection and Use of Chicken Bone Marrow-Derived Macrophages (BMDMs)

Rhode Island Red chickens of mixed sex were raised in a specific pathogen-free environment at the Pirbright Institute until aged between two and three weeks old and weighing between 200 and 300 g. Femurs were collected following euthanasia under project licence number P6257C392, approved by the UK Home Office and The Pirbright Institute Ethical Committee on 10 August 2020 at The Pirbright Institute. The use of the tissues in the assays described here was also approved by the University of Surrey’s Animal Welfare and Ethical Review Body (AWERB) (reference OUT085 (approved 10 February 2023) and OUT092 (approved 14 July 2025)).

### 2.5. Isolation and Differentiation of Chicken Bone Marrow-Derived Macrophages (BMDMs)

Chicken bone marrow-derived macrophages (BMDMs) were generated by the cutting of chicken femurs short ways and flushing the bone marrow with 2% FBS in PBS with a 27-gauge needle. A single cell suspension was achieved by mashing cells through a 40-µm-pore-size Falcon cell strainer (BD Biosciences, Oxford, UK). Cell suspension was then pelleted by centrifugation at 500× *g* for 5 min at 4 °C before resuspension in 10 mL 2% FBS in PBS. Following this, cell suspension was overlayed with Histopaque 1.119 (Merck, Kenilworth, UK) and centrifuged for 30 min at 500× *g* and 4 °C. The buffy coat was then isolated, washed with 20 mL 2% FBS in PBS and resuspended in BMDM media (RPMI 1640 supplemented with 5% FBS, 5% CS, 1% sodium pyruvate, and 1% pen-strep (Merck, Kenilworth, UK)). Cells were then seeded at a density of 2 × 10^6^ cells in T75 flasks at 41 °C + 5% CO_2_ in BMDM media supplemented with 2% macrophage colony stimulating factor 1 (mCSF-1) expressed and produced in our laboratories with fresh media and growth factors added on days 1, 3, and 5.

### 2.6. Preparation of Sodium Butyrate for Use in Cell Culture Assays

The sodium butyrate was prepared by dissolving 0.11 g of sodium butyrate powder (cat no A11079.22, ThermoFisher Scientific, Basingstoke, UK) in 5 mL sterile double distilled water, before sterile filtration with a 0.45 µm filter to produce a 200 mM/mL stock. This stock was then diluted in RPMI + 5% FBS + 5% CS to the desired concentrations in cell culture media.

### 2.7. Priming of HD11 Cells with Sodium Butyrate

The HD11 cells were seeded in 24-well plates at 2 × 10^5^ cells/mL in RPMI 1640 medium with GlutaMAX 5% FBS, 5% CS and incubated for 24 h at 41 °C + 5% CO_2_. Following incubation, for each biological replicate 12 replica wells were exposed to either 1 mM sodium butyrate (prepared as described above) or a PBS as a control. Cells were then incubated for a further 24 h prior to use in assays.

### 2.8. Priming of BMDMs with Sodium Butyrate

During differentiation of BMDMs as described above, cells were primed using a modified approach based on the previous studies by Verwoolde et al. and Schulthess et al. [5,29]. Cell culture media was supplemented with sodium butyrate 24 h after generation on day 1. Cell culture media was refreshed with fresh sodium butyrate and growth factors on days 3 and 5. On day 5, media was aspirated, and the cells were washed twice with pre-warmed PBS. After washing, PBS was removed and 5 mL TrypLE express (ThermoFisher Scientific, Basingstoke, UK) was added and incubated at 37 °C for 3 min before cells were detached using a sterile cell scraper, with detachment confirmed by microscopy. Enzymatic activity was quenched by the addition of 10 mL BMDM cell culture media and cells pelleted by centrifugation at 500× *g* for 5 min. After discarding the supernatant, the cell pellet was resuspended in BMDM media and enumerated with exclusion of dead cells by Trypan blue staining (Fisher, Basingstoke, UK) and counted with a Neubauer haemocytometer (Marienfeld, Germany). BMDMs were seeded in tissue-culture-treated plates in antibiotic-free BMDM culture media.

### 2.9. Determination of HD11 or BMDM Cell Viability Following Butyrate Priming and Bacterial Challenge

Triplicate wells of cells which had undergone priming with sodium butyrate or incubation with media alone were challenged with APEC O78:ST23 at a MOI of 10 (2 × 10^6^ CFU/mL) or a PBS mock as a control using a protocol modified from that described by Schulthess et al. Plates were centrifuged at 4 °C for 3 min at 300× *g* before incubation at 41 °C + 5% CO_2_ for 1 h [29,30]. After the addition of cell culture media containing 100 µg/mL gentamicin, cells were incubated for two hours at 41 °C + 5% CO_2_ before the removal of the supernatant and washing of cells twice with warm PBS. To each well, 300 µL of 0.25% trypsin was added, and plates were incubated at 37 °C for 4 min to detach cells. Trypsin activity was quenched by the addition of 300 µL cell culture media and mixed by pipetting up and down to detach remaining cells. Following this, 20 µL from each well was transferred to a microcentrifuge tube containing 20 µL of trypan blue. After gentle mixing by pipetting up and down, 10 µL of the cell suspension was transferred to a Neubauer haemocytometer where live (white) and dead (blue) cells were enumerated.

### 2.10. Determination of Bacterial Intracellular Survival

HD11 or BMDMs with and without priming with sodium butyrate were seeded in 24-well tissue-culture-treated plates at 2 × 10^5^ cell/mL in a total of 1 mL media per well for least 24 h prior to assay to ensure adherence. Triplicate wells were challenged with bacterial cultures at a MOI of 10. Plates were centrifuged at 4 °C for 3 min at 300× *g* before incubation at 41 °C + 5% CO_2_ for 1 h. Supernatant was then removed and replaced with 1 mL of RPMI + 5% FBS + 5% CS + 100 µg/mL gentamicin. Cells were then incubated at 41 °C, with 5% CO_2_ for two hours before removal of supernatant. The cells were washed twice with prewarmed PBS, and 1 mL of PBS + 1% triton X-100 (Merck, Kenilworth, UK) was added and agitated through pipetting to disrupt the eukaryotic cells. Bacterial intracellular survival was determined using the Miles and Misra technique [27]. Briefly, cell lysate was serial diluted 10-fold in sterile PBS and 20 µL of each dilution was dropped in triplicate onto nutrient agar before incubation for 16 h at 37 °C, aerobically. Colonies were then counted and colony-forming units (CFU) per mL calculated.

### 2.11. Bacterial Growth Kinetics

A single representative colony from each bacterial isolate was used to inoculate 10 mL of fresh LB broth in a 50 mL Falcon tube before aerobic incubation at 37 °C, with shaking at 225 RPM, for 18 h. Following incubation, 100 μL was removed and used to inoculate 9.9 mL LB in a 50 mL centrifuge tube before growth for two hours at 37 °C, aerobically, with shaking at 225 RPM. Cultures were pelleted by centrifugation at 5000× *g* for 10 min and media was discarded before resuspension in RPMI cell culture media supplemented with 5% FBS and 5% CS with or without a final concentration of 1 mM sodium butyrate at a 0.5 MacFarland standard. Bacterial cultures were added to triplicate wells of a 96-well plate before transferring to a TECAN SPARK plate reader (Tecan Group Ltd., Männedorf, Switzerland) pre-incubated to 37 °C with OD_600_ recorded every 15 min.

### 2.12. Bacterial Adhesion and Internalisation

HD11 or BMDMs were seeded in duplicate 24-well tissue-culture-treated plates at 2 × 10^5^ cell/mL in a total of 1 mL media per well at least 24 h prior to assay to ensure adherence. Concurrently, a single representative APEC O78:ST23 (SAP503) colony was used to inoculate 10 mL of fresh LB broth in a 50 mL Falcon tube before aerobic incubation at 37 °C, with shaking at 225 RPM, for 18 h. Following incubation, 100 μL of bacterial culture was removed and used to inoculate 9.9 mL LB broth or 9.9 mL LB broth supplemented with 1 mM butyrate in a 50 mL centrifuge tube before growth for two hours at 37 °C, aerobically, with shaking at 225 RPM. Triplicate wells of HD11 or BMDM cells in duplicate plates were challenged with a MOI of 10 of APEC grown with or without 1 mM sodium butyrate supplementation before centrifugation at 4 °C for 3 min at 300× *g*. To determine association, challenged HD11 or BMDM cells were incubated at 41 °C + 5% CO_2_ for two hours before aspiration of the supernatant and washing twice with warm PBS before the addition of 1 mL 1% triton X in PBS to lyse cells. To determine internalisation, challenged HD11 or BMDM cells were incubated at 41 °C + 5% CO_2_ for two hours before aspiration of the supernatant and the addition of 1 mL cell culture media supplemented with 100 µg/mL gentamicin and incubation for an additional two hours. Cell supernatant was then aspirated and HD11 or BMDMs were washed twice with warm PBS and 1 mL 1% triton X in PBS was added to lyse cells. Bacterial viability within the cell lysate was then quantified using the Miles and Misra technique [27]. Adhesion was determined by subtraction of averaged internalisation CFU/mL from calculated association CFU/mL.

### 2.13. Quantification of Macrophage Phagocytosis of GFP-Tagged Inactivated E. coli K12

HD11 or BMDMs with and without priming with sodium butyrate were seeded in triplicate wells in 96-well tissue-culture-treated plates at 2 × 10^5^ cell/mL in a total of 100 µL media per well at least 24 h prior to assay to ensure adherence. Cell culture media was then aspirated, and cells were incubated with 100 µL of the Vybrant Phagocytosis assay (Product code. V6694) GFP-tagged K12 bioparticles (ThermoFisher, Paisley, UK) prepared as per the manufacturer’s instructions for 2 h at 41 °C + 5% CO_2_. The supernatant was then removed and 100 uL 1× trypan blue was added to the cells for 1 min before its removal and the measurement of the GFP fluorescence signal at excitation 488 nm and emission at 510 nm using a TECAN SPARK plate reader (Tecan Group Ltd., Männedorf, Switzerland).

### 2.14. Determination of Nitric Oxide and Reactive Oxygen Species Production

HD11 or BMDMs, either primed or unprimed with sodium butyrate, were seeded in 24-well tissue-culture-treated plates at 2 × 10^5^ cell/mL in a total of 1 mL media per well at least 24 h prior to assay to ensure adherence. Triplicate wells were challenged with bacterial isolates at a MOI of 10 were centrifuged at 4 °C for 3 min at 300× *g* before incubation at 41 °C + 5% CO_2_ for 1 h.

To determine nitric oxide (NO) production, cell culture supernatant was removed and replaced with 1 mL of RPMI + 5% FBS + 5% CS + 100 µg/mL gentamicin and incubated for a further four hours. The cell supernatant was transferred to a 1.5 mL microcentrifuge tube and centrifuged at 2500× *g* for 10 min at 4 °C to pellet cell debris. Following this, 150 µL of the supernatant was mixed with 130 µL ddH_2_O and 20 µL Griess reagent (ThermoFisher, Paisley, UK) in triplicate in a 96-well plate (Greiner, Stonehouse, UK). The cells were incubated at room temperature, protected from light for 30 min, absorbance was read at 548 nm on a TECAN Spark plate reader, and nitrate concentration (the autooxidation product of NO) was determined using a calibration curve.

For detection of ROS production, the cell culture supernatant was removed and replaced with 1 mL of RPMI + 5% FBS + 5% CS + 100 µg/mL gentamicin + 20 µM DCFA (D399, ThermoFisher, Paisley, UK). The cells were incubated in the Clariostar plate reader (BMG Labtech, Ortenberg, Germany) at 41 °C + 5% CO_2_ for a further 10 hours with fluorescence at 495 nm excitation and 529 nm emission measured every 15 min.

### 2.15. Determination of Bacterial Intracellular Killing in Inhibitor Treated Chicken Macrophages

HD11 or BMDMs with and without priming with sodium butyrate were seeded in 24-well tissue-culture-treated plates at 2 × 10^5^ cell/mL in a total of 1 mL media per well at least 24 h prior to the assay to ensure cell adherence. Filter-sterilised chloroquine diphosphate salt (2.5 µM) (Product code. C6628, Merck, Kenilworth, UK), rapamycin (10 µg/mL) (Product code. 553210, Merck, Kenilworth, UK), N-acetyl-L-cysteine (0.5 mM) (Product code. A9165, Merck, Kenilworth, UK), or a PBS blank was added to triplicate wells of HD11 cells or BMDMs. Cells were then incubated for 24 h before being challenged with SAP 503 (APEC O78:ST23) at a MOI of 10. Plates were centrifuged at 4 °C for 3 min at 300× *g* before incubation at 41 °C + 5% CO_2_ for 1 h. The supernatant was then removed and replaced with 1 mL of RPMI + 5% FBS + 5% CS + 100 µg/mL gentamicin + 2.5 µM chloroquine diphosphate or PBS blank. Cells were then incubated at 41 °C with 5% CO_2_ for two hours before the removal of the supernatant. The cells were washed twice with prewarmed PBS and 1 mL of PBS + 1% triton was added and agitated through pipetting to disrupt the eukaryotic cells. Bacterial intracellular survival was determined using the Miles and Misra technique [27].

### 2.16. Transfection of HD11 Cells with EGFP-Tagged avLC3B and Quantification of LC3B Accumulation

HD11 cells seeded in 24-well tissue-culture-treated plates at 2 × 10^5^ cell/mL in a total of 1 mL media were transfected with GFP-avLC3B plasmid DNA (A gift from Dr. Helena Maier; The Pirbright Institute, Pirbright, UK [31]) using lipofectamine 2000 (ThermoFisher, Paisley, UK) as per the manufacturer’s instructions. Cells were incubated for 24 h at 41 °C + 5% CO_2_ prior to the addition of 1 mM butyrate or PBS blank to 12 replica wells, followed by the addition of 2.5 µM chloroquine diphosphate, 10 µg/mL rapamycin, 50 µg/mL L-leucine, or PBS blank to triplicate EGFP-avLC3 transfected wells. Cells were incubated for a further 24 h at 41 °C + 5% CO_2_ before the quantification of LC3B accumulation by measurement of EGFP fluorescence at excitation 488 nm and emission at 510 nm using a TECAN Spark plate reader.

### 2.17. RNA Extraction and One-Step RT-qPCR Analysis

Total RNA was isolated from HD11 cells and BMDMs both primed with sodium butyrate and a PBS control using an RNA extraction kit (New England Biolabs, T2010S, Ipswich, MA, USA) according to the manufacturer’s instructions. RNA quality and concentration was determined using a Biodrop spectrophotometer (DKSH, Zurich, Switzerland) and samples were stored at −80 °C. Expression of *LC3*, *ATG16L1*, *TOR*, *RHO*, and *28S* (Table 2), was determined by the transfer of 1.5 µL RNA to a 96-well PCR plate containing 5 µL Luna^®^ Universal One-Step Reaction Mix, 0.5 µL Luna^®^ WarmStart^®^ RT Enzyme Mix (New England Biolabs, E3005S, Ipswich, MA, USA), 0.5 µL forward primer, 0.5 µL reverse primer, and 2 µL nuclease free water. qPCR analysis was performed using a BioRad CFX96 RealTime system and C1000 Touch thermal cycler (BioRad, Hercules, CA, USA) as per the manufacturer’s instructions. Single amplification products were confirmed with melt curves and relative expression was calculated using the 2^−ΔΔCt^ method [32].

### 2.18. Statistical Analysis

Significance was determined for experiments involving grouped comparisons of three or more groups using the Kruskal–Wallis statistical test [36] and Dunn’s test of multiple comparisons [37] unless otherwise stated. For experiments with two groups, significance was determined by unpaired Student’s *t*-test unless otherwise stated. For kinetic assays, area under the curve (AUC) analysis followed by the Kruskal–Wallis statistical test [36] and Dunn’s test of multiple comparisons [37] was performed to determine significance. All statistical analyses were performed using GraphPad Prism software (v. 9.1.2, GraphPad Software Inc., San Diego, CA, USA).

## 3. Results

### 3.1. Butyrate Priming Enhances Antimicrobial Activity of Chicken Macrophage-like HD11 Cells Against Diverse Bacterial Pathogens

**Figure 1:**
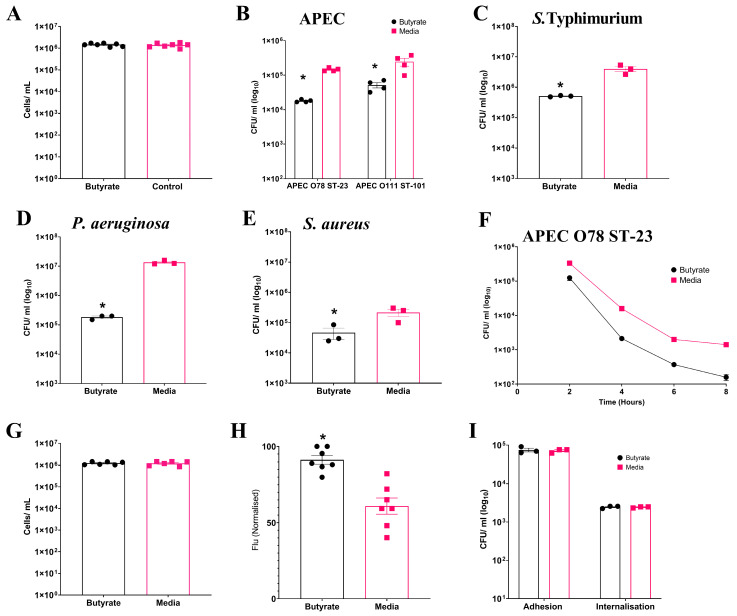
Priming of HD11 cells with sodium butyrate reduces intracellular survival of multiple bacterial pathogens. (**A**) HD11 cell viability following 24 h exposure to 1 mM sodium butyrate. Intracellular survival of (**B**) APEC O78 ST-23 (*n* = 4) and APEC O111 ST-101 (*n* = 3), (**C**) *S.* Typhimurium (*n* = 3), (**D**) *P. aeruginosa* (*n* = 3), and (**E**) *S. aureus* (*n* = 3) in butyrate-primed and media-only cells following gentamicin protection assays. (**F**) Intracellular survival of APEC O78 ST-23 in butyrate-primed or media-only HD11 cells at multiple timepoints following infection (*n* = 3). (**G**) Viability of butyrate-primed or media-only HD11 cells following challenge with APEC O78 ST-23 (*n* = 6). (**H**) Phagocytosis of GFP-tagged inactivated K12 *E. coli* by butyrate-primed or media-only macrophages (*n* = 7). (**I**) Adhesion and internalisation of APEC O78 ST-23 grown with and without butyrate-supplemented cell culture media to and within HD11 cells (*n* = 3). Experiments were independently performed 3–7 (*n*) times in triplicate. Data presented as mean ± SEM. Significance was determined between two groups by Welch’s *t*-test and Kruskal–Wallis statistical test and Dunn’s test of multiple comparisons for three groups or more * = *p* ≤ 0.05.

Having analysed the effects of sodium butyrate on cell viability as described in Section 2, we established that concentrations up to 1 mM were non-toxic to HD11 cells. To investigate the impact of sodium butyrate priming on chicken macrophages, we treated the immortalised chicken macrophage-like cell line HD11 with a non-toxic concentration of 1 mM sodium butyrate for 24 h (Figure S1), as depicted in Figure 1A (Average: Butyrate: 1.45 × 10^6^ ± 8.8 × 10^4^ cells/mL, Media: 1.39 × 10^6^ ± 1.16 × 10^5^ cells/mL). The effect of sodium butyrate priming on the intracellular survival of multiple bacterial pathogens within HD11 cells was investigated using gentamicin protection assays. Significant reductions (*p* ≤ 0.05) in bacterial intracellular survival were observed following the challenge of sodium butyrate-primed HD11 cells with the Gram-negative APEC O78 ST-23 (Average ± SEM: Butyrate: 1.8 × 10^4^ ± 787 CFU/mL, Media: 1.45 × 10^5^ ± 8.1 × 10^3^ CFU/mL, Figure 1B), APEC O88 ST-101 (Average ± SEM: Butyrate: 5.1 × 10^4^ ± 8.8 × 10^3^ CFU/mL, Media: 2.44 × 10^5^ ± 6.1 × 10^4^ CFU/mL, Figure 1B), *Salmonella* Typhimurium (Average ± SEM: Butyrate: 5 × 10^5^ ± 1.4 × 10^4^ CFU/mL, Media: 4 × 10^6^ ± 7.6 × 10^5^ CFU/mL, Figure 1C), and *Pseudomonas aeruginosa* (Average ± SEM: Butyrate: 1.83 × 10^5^ ± 1.6 × 10^4^ CFU/mL, Media: 1.35 × 10^7^ ± 1.25 × 10^6^ CFU/mL, Figure 1D), as well as the Gram-positive *Staphylococcus aureus* (Average ± SEM: Butyrate: 4.6 × 10^4^ ± 1.9 × 10^4^ CFU/mL, Media: 2.16 × 10^5^ ± 6 × 10^4^ CFU/mL, Figure 1E). Reduced bacterial survival was observed within 8 h post infection with APEC (Figure 1F) with significant reductions in bacterial survival at all timepoints (*p* ≥ 0.05). This trend was observed at 10 and 18 h post infection, but bacterial levels for butyrate-primed intracellular survival were below the limit of detection.. The reductions in bacterial intracellular survival following butyrate priming were confirmed to not be the result of reduced HD11 viability following infection, as no significant differences in cell numbers were observed following infection and incubation with media containing gentamicin (*p* ≥ 0.05) (Average ± SEM: Butyrate: 1.2 × 10^6^ ± 8 × 10^4^ cells/mL, Media: 1.2 × 10^6^ ± 1.1 × 10^5^ cells/mL, Figure 1G). The reductions in bacterial survival were also not due to impaired phagocytic activity due to the butyrate treatment of the macrophages. Instead, the phagocytosis of inactivated GFP-tagged K12 *E. coli* was significantly enhanced in butyrate-primed HD11 cells (*p* ≤ 0.05) (Average ± SEM: Butyrate: 91.3% ± 7.4%, Media: 60.9% ± 14.1%, Figure 1H). The presence of butyrate at concentrations of 1 mM within the cell culture media and cell environment was also observed to have no significant impact on bacterial growth kinetics, suggesting (*p* ≥ 0.05) (Figure S2), and growth in the presence of butyrate had no significant effect on the adhesion or internalisation of APEC O78 ST-23 in HD11 cells (*p* ≥ 0.05) (Figure 1I). Collectively, these findings suggest that priming of HD11 chicken macrophage-like cells by exposure to butyrate results in an enhanced antimicrobial response against the bacterial pathogens.

### 3.2. Butyrate Priming During Differentiation Enhances Antibacterial Activity of Primary Chicken BMDM

**Figure 2:**
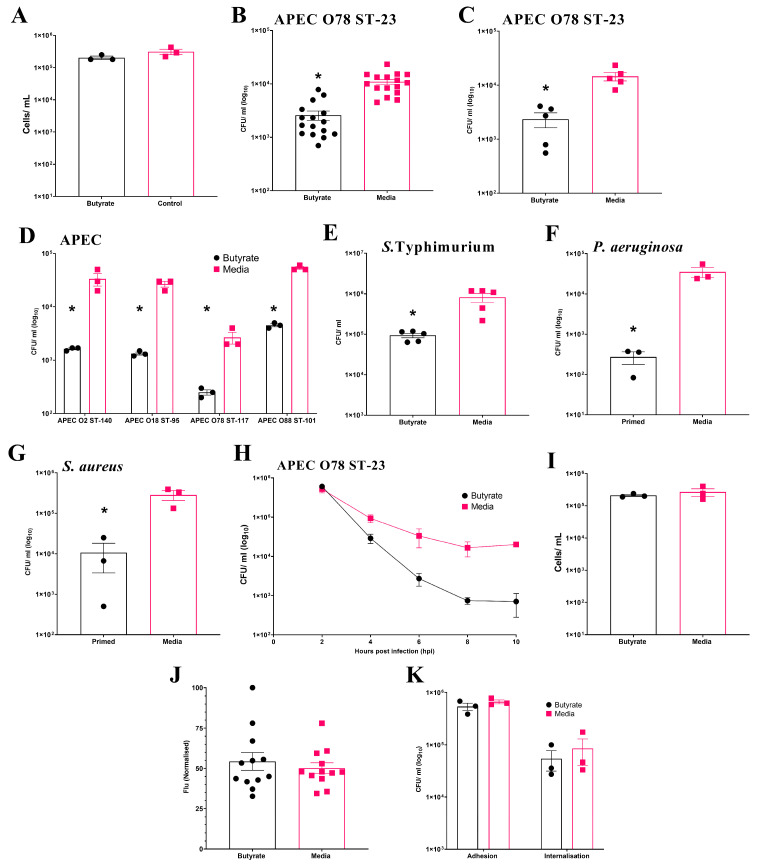
Priming of chicken BMDM cells with butyrate during differentiation reduces intracellular survival of multiple bacterial pathogens. (**A**) BMDM cell viability following exposure to 1 mM butyrate during differentiation (*n* = 3). Bacterial intracellular survival of APEC O78 ST-23 in BMDMs primed with sodium butyrate or media alone generated from (**B**) three birds with sixteen technical replicates (*n* = 16) and (**C**) five individual birds in triplicate (*n* = 5). (**D**) Intracellular survival in BMDMs primed with sodium butyrate or media alone of APEC O2 ST-140, APEC O18 ST-95, APEC O78 ST-117, APEC O88 ST-101 (*n* = 3). Intracellular survival of (**E**) *S.* Typhimurium (*n* = 3), (**F**) *P. aeruginosa* (*n* = 3), and (**G**) *S. aureus* (*n* = 3), following gentamicin protection assays with butyrate-primed and media only BMDMs isolated from three pooled chicken femurs. (**H**) Intracellular survival of APEC O78 ST-23 in butyrate-primed or media-only BMDMs at multiple timepoints following infection (*n* = 3). (**I**) Viability of butyrate-primed or media-only HD11 cells following challenge with APEC O78 ST-23 (*n* = 3). (**J**) Phagocytosis of GFP-tagged inactivated K12 *E. coli* by butyrate-primed or media-only BMDMs (*n* = 12). (**K**) Adhesion and internalisation of APEC O78 ST-23 grown with and without butyrate-supplemented cell culture media to and within BMDMs (*n* = 3). Experiments performed with pooled BMDMs generated from three birds in triplicate, unless otherwise stated. Data presented as mean ± SEM. Significance was determined between two groups by Welch’s *t*-test and Kruskal–Wallis statistical test and Dunn’s test of multiple comparisons for three groups or more * = *p* ≤ 0.05.

Initially, the effect of butyrate exposure on cell viability during chicken BMDM differentiation was examined. The results show no significant (*p* ≥ 0.05) effect on the number of viable cells at the end of differentiation (Average ± SEM: Butyrate: 2 × 10^5^ ± 2.3 × 10^4^ cells/mL, Media: 3.1 × 10^5^ ± 6.1 × 10^4^ cells/mL, Figure 2A). In the initial experiment using BMDMs pooled from three individual birds, intracellular survival of APEC O78 ST-23 was significantly reduced (*p* ≤ 0.05) following sodium butyrate priming (Average ± SEM: Butyrate: 2.6 × 10^3^ ± 5.1 × 10^2^ CFU/mL, Media: 1.1 × 10^4^ ± 1.2 × 10^3^ CFU/mL, Figure 2B). This reduction was confirmed in a follow-up experiment using primed BMDMs isolated from five individual birds (Average ± SEM: Butyrate: 2.3 × 10^3^ ± 7.20 × 10^2^ CFU/mL, Media: 1.45 × 10^4^ ± 2.5 × 10^3^ CFU/mL, Figure 2C). Beyond APEC O78 ST-23, priming of pooled BMDMs from three different birds also led to significant reductions (*p* ≤ 0.05) in the bacterial intracellular survival of multiple APEC lineages (Figure 2D). Moreover, butyrate priming also led to a significantly reduced bacterial survival of *S.* Typhimurium (Average ± SEM: Butyrate: 9.3 × 10^4^ ± 1.2 × 10^4^ CFU/mL, Media: 8.18 × 10^5^ ± 2.06 × 10^5^ CFU/mL, Figure 2E), *P. aeruginosa* (Average ± SEM: Butyrate: 272 ± 94 CFU/mL, Media: 3.5 × 10^4^ ± 9.87 × 10^3^ CFU/mL, Figure 2F), and *S. aureus* (Average ± SEM: Butyrate: 1.1 ± 7.3 × 10^3^ CFU/mL, Media: 2.8 × 10^5^ ± 7.8 × 10^4^ CFU/mL, Figure 2G). However, priming of BMDMs with sodium butyrate after differentiation had no significant effect on bacterial intracellular survival (Average ± SEM: Butyrate: 2 × 10^4^ ± 2.8 × 10^3^ CFU/mL, Media: 1 × 10^4^ ± 2.8 × 10^3^ CFU/mL, Figure S3), suggesting that modulation of macrophages can only occur during their differentiation from bone marrow progenitor cells. In contrast to HD11 cells, where bacterial survival was reduced as early as 2 h post-challenge, reductions in bacterial survival in butyrate-primed BMDMs were not observed until 4 h post-infection. However, once established, the reduction in bacterial survival within butyrate-primed BMDMs was sustained across all subsequent timepoints examined (Figure 2H). Cell viability was also confirmed to be comparable between butyrate-primed and media-only BMDMs (Average ± SEM: Butyrate: 2.1 × 10^5^ ± 1.5 × 10^4^ cells/mL, Media: 2.66 × 10^5^ ± 7 × 10^4^ cells/mL, Figure 2I), supporting that the observed reductions in bacterial survival were not due to decreased cell numbers. Unlike butyrate-primed HD11 cells, no significant difference in phagocytosis of GFP-tagged K12 *E. coli* was observed within primed BMDMs (*p* ≥ 0.05) (Average ± SEM: Butyrate: 54.3% ± 5.5%, Media: 50.1% ± 3.4%, Figure 2J), supporting that reduced bacterial survival is not a result of impaired phagocytosis in butyrate-primed BMDMs. The presence of butyrate in growth media was also found to have no impact on the ability of bacteria to adhere to or become internalised by BMDMs (*p* ≥ 0.05). Taken alongside the observations described within the HD11 cells, this collectively suggests that immune priming with butyrate enhances the antibacterial activity of chicken macrophages.

### 3.3. Enhancement of Chicken Macrophage Antimicrobial Activity Following Butyrate Priming Is Dependent upon Increased ROS Production

**Figure 3:**
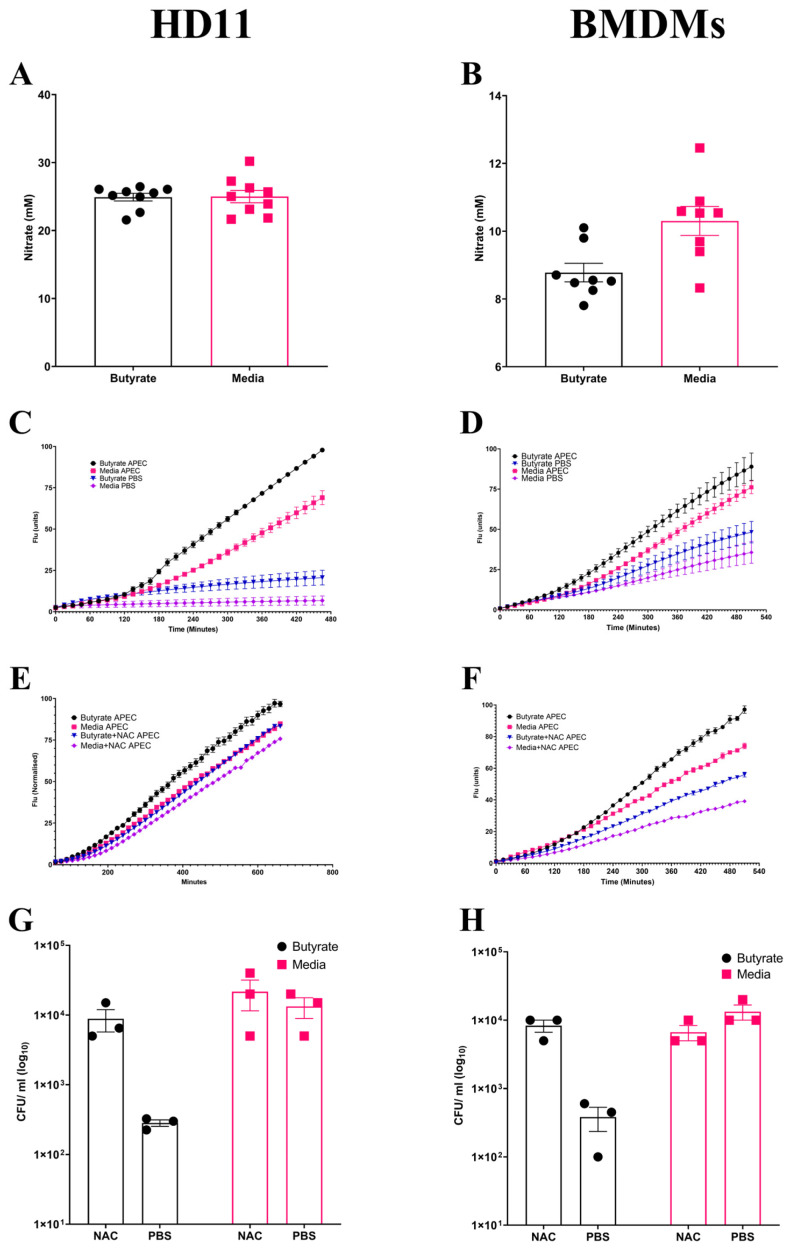
Priming of chicken macrophages increases reactive oxygen species production which is associated with enhanced bacterial killing. Nitric oxide production of butyrate-primed and media-only (**A**) HD11 cells (*n* = 8), (**B**) BMDMs (*n* = 7) four hours post-challenge with APEC O78 ST-23 determined by Griess assay. (**C**) HD11 and (**D**) BMDMs reactive oxygen species production of APEC O78 ST-23- and PBS blank-challenged butyrate-primed and media-only cells determined by DCFA assay (*n* = 3). (**E**) HD11 and (**F**) BMDMs reactive oxygen species production of APEC O78 ST-23- and PBS blank-challenged butyrate-primed and media-only cells with and without the addition of 0.5 mM NAC determined by DCFA assay (*n* = 3). (**G**) HD11 and (**H**) bacterial intracellular survival of APEC O78 ST-23 in butyrate-primed and media-only cells with and without 0.5 mM NAC determined by gentamicin protection assay (*n* = 3). Experiments independently performed 3–4 times (*n*) in triplicate. BMDMs generated from three individual birds and pooled. Data presented as mean ± SEM. Significance was determined for experiments involving grouped comparisons of three or more groups using the Kruskal–Wallis statistical test and Dunn’s test of multiple comparison. For experiments with two groups, significance was determined by Welch’s *t*-test. For kinetic assays, significance was determined by area under the curve (AUC) analysis followed by Kruskal–Wallis statistical test and Dunn’s test of multiple comparisons.

Following observations suggesting the enhancement of antimicrobial activity by butyrate priming of chicken macrophages, the potential mechanism(s) for the increased antimicrobial response was investigated. NO is frequently used as an indicator of the magnitude of inflammatory immune response, functioning as an antimicrobial agent, and is suggestive of a strong inflammatory response. No significant changes (*p* ≥ 0.05) in NO production were observed in butyrate-primed HD11 cells (Average ± SEM: Butyrate: 24.9 ± 0.5 mM, Media: 24.9 ± 0.9 mM, Figure 3A) or BMDMs (Average ± SEM: Butyrate: 8.7 ± 0.5 mM, Media: 10.3 ± 0.4 mM, Figure 3B) following APEC O78 ST-23 challenge. This indicates that reduced bacterial survival is not associated with NO production. Alongside NO synthesis, the production of ROS derived from NADPH-oxidase composes the antibacterial mechanisms within macrophages. ROS production within butyrate-primed and control (media-only) cells was investigated following APEC O78 ST-23 challenge. Butyrate priming led to a significant (*p* ≤ 0.05) increase in ROS production following challenge compared to the control HD11 cells (Figure 3C) or BMDMs (Figure 3D). Interestingly, ROS production was significantly increased in unchallenged butyrate-primed HD11 (Figure 3C) and BMDMs (Figure 3D) (*p* ≤ 0.05) when compared to cells cultured in media alone. To determine the role of ROS production in the enhancement of antimicrobial activity in butyrate-primed chicken macrophages, the ROS scavenger N-acetyl-L-cystine (NAC) was utilised. Addition of 0.5 mM NAC resulted in reduced ROS production in both butyrate-primed and control HD11 cells (Average ± SEM: Butyrate: 2 × 10^2^ ± 30 CFU/mL, Butyrate + NAC: 8.8 × 10^3^ ± 3.1 × 10^3^ CFU/mL, Media: 1.3 × 10^4^ ± 4.4 × 10^3^ CFU/mL, Media + NAC: 2.1 × 10^4^ ± 1 × 10^4^ CFU/mL, Figure 3E) and BMDMs (Average ± SEM: Butyrate: 3.8 × 10^2^ ± 1.4 × 10^2^ CFU/mL, Butyrate + NAC: 8.3 × 10^3^ ± 1.6 × 10^3^ CFU/mL, Media: 1.3 × 10^4^ ± 3.3 × 10^3^ CFU/mL, Media + NAC: 6.6× 10^3^ ± 1.6 × 10^3^ CFU/mL, Figure 3F). The presence of NAC was found to attenuate butyrate-priming-associated reductions in intracellular survival of APEC O78 ST-23 in both HD11 cells (Figure 3G) and BMDMs (Figure 3H). The results demonstrate that there is no significant difference in bacterial survival between NAC treated butyrate-primed and media only control macrophages (*p* ≥ 0.05). Collectively, these findings suggest that butyrate priming of both the immortalised HD11 cell line and primary chicken BMDMs leads to increased ROS production, both at rest and in response to bacterial challenge. Moreover, inhibition of ROS accumulation by NAC attenuates the enhanced antimicrobial activity associated with butyrate priming, indicating this enhanced response is ROS-dependent.

### 3.4. Butyrate-Induced Autophagy Enhances Bacterial Clearance in Chicken Macrophages

**Figure 4:**
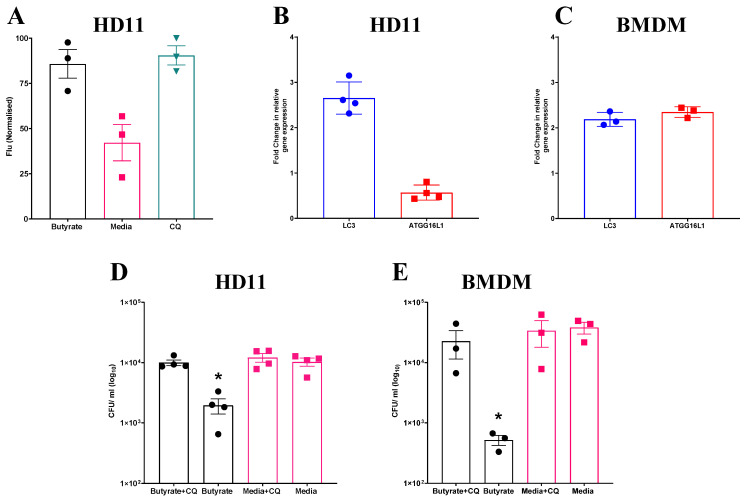
Increased accumulation of avLC3B in primed HD11 cells and attenuation of priming-associated antimicrobial activity following exposure to chloroquine suggests a mechanistic role for autophagy. (**A**) Fluorescence intensity of HD11 cells transfected with avLC3B-EGFP prior to priming with 1 mM sodium butyrate, exposure to 1.5 µM CQ, or media alone for 24 h. GFP-avLC3B accumulation was quantified by measurement of GFP fluorescence at excitation 488 nm and emission at 510 nm (*n* = 3). Fold change in autophagy-associated genes LC3 and ATG16L1 in (**B**) primed HD11 cells (*n* = 4) and (**C**) primed BMDMs relative to PBS-only controls (*n* = 3). Bacterial intracellular survival of APEC O78 ST-23 in butyrate-primed and media-only cells with and without 1.5 µM CQ determined by gentamicin protection assay in (**D**) HD11 cells (*n* = 4) and (**E**) BMDMs (*n* = 3). Experiments independently performed 2–4 times (*n*), in triplicate. BMDMs generated from femurs of three individual birds and pooled. Data presented as mean ± SEM. Significance was determined for experiments involving grouped comparisons of three or more groups using the Kruskal–Wallis statistical test and Dunn’s test of multiple comparison. For experiments with two groups, significance was determined by Welch’s *t*-test. * = *p* ≤ 0.05.

Given the observed increase in ROS production in both HD11 cells and BMDMs primed with butyrate, and the apparent dependence of intracellular bacterial elimination on this increase, further investigation into associated cellular functions was conducted. One pathway which has been observed to be regulated by, and able to regulate ROS production is autophagy [38]. To determine whether butyrate priming of chicken macrophages induces autophagy, HD11 cells were transfected with an avLC3B-EGFP plasmid to determine LC3 accumulation. HD11 cells primed with butyrate, as well as those treated with chloroquine (CQ), which blocks autophagosome and lysosome fusion and was used as a positive control for LC3 accumulation, saw significantly increased fluorescence intensity compared to media-only HD11 cells (*p* ≤ 0.05) (Average ± SEM: CQ: 90.4% ± 5.3% Butyrate: 85.7% ± 7.9%, Media: 42.1% ± 9.9%, Figure 4A). Furthermore, RT-qPCR analysis revealed expression of LC3 gene was increased 2-fold in both primed HD11 cells (Figure 4B) and BMDMs (Figure 4C), while ATG16L1 gene expression was only increased in BMDMs. Collectively, the findings indicate that butyrate priming leads to the upregulation of autophagy-associated genes and increased LC3 expression in chicken macrophages.

To investigate the involvement of autophagy in the antibacterial responses induced by butyrate priming, primed HD11 cells or BMDMs were exposed to CQ, a lysosomotropic agent used to inhibit autophagy, prior to being challenged with APEC O78 ST-23. Subsequently, bacterial intracellular survival was quantified via a gentamicin protection assay. CQ attenuated enhanced antimicrobial activity in both butyrate-primed HD11 cells (Average ± SEM: Butyrate: 1.9 × 10^3^ ± 5.4 × 10^2^ CFU/mL, Butyrate + CQ: 1 × 10^4^ ± 1 × 10^3^ CFU/mL, Media: 1 × 10^4^ ± 1.5 × 10^3^ CFU/mL, Media + CQ: 1.2 × 10^4^ ± 1.5 × 10^3^ CFU/mL, Figure 4D) and BMDMs (Average ± SEM: Butyrate: 5.1 × 10^2^ ± 98 CFU/mL, Butyrate + CQ: 2.2 × 10^4^ ± 1.1 × 10^4^ CFU/mL, Media: 3.8 × 10^4^ ± 8.4 × 10^3^ CFU/mL, Media + CQ: 3.3 × 10^4^ ± 1.5 × 10^4^ CFU/mL, Figure 4E), with bacterial intracellular survival returning to levels equivalent to those in the control cells (*p* ≥ 0.05). Taken together, these results indicate that butyrate priming induces increased autophagy, as demonstrated by LC3 accumulation and the observed reduction in enhanced antimicrobial activity upon CQ treatment in primed HD11 cells and BMDMs.

### 3.5. The Role of mTOR/Autophagy in Butyrate Priming’s Enhancement of Chicken Macrophage Antimicrobial Activity

**Figure 5:**
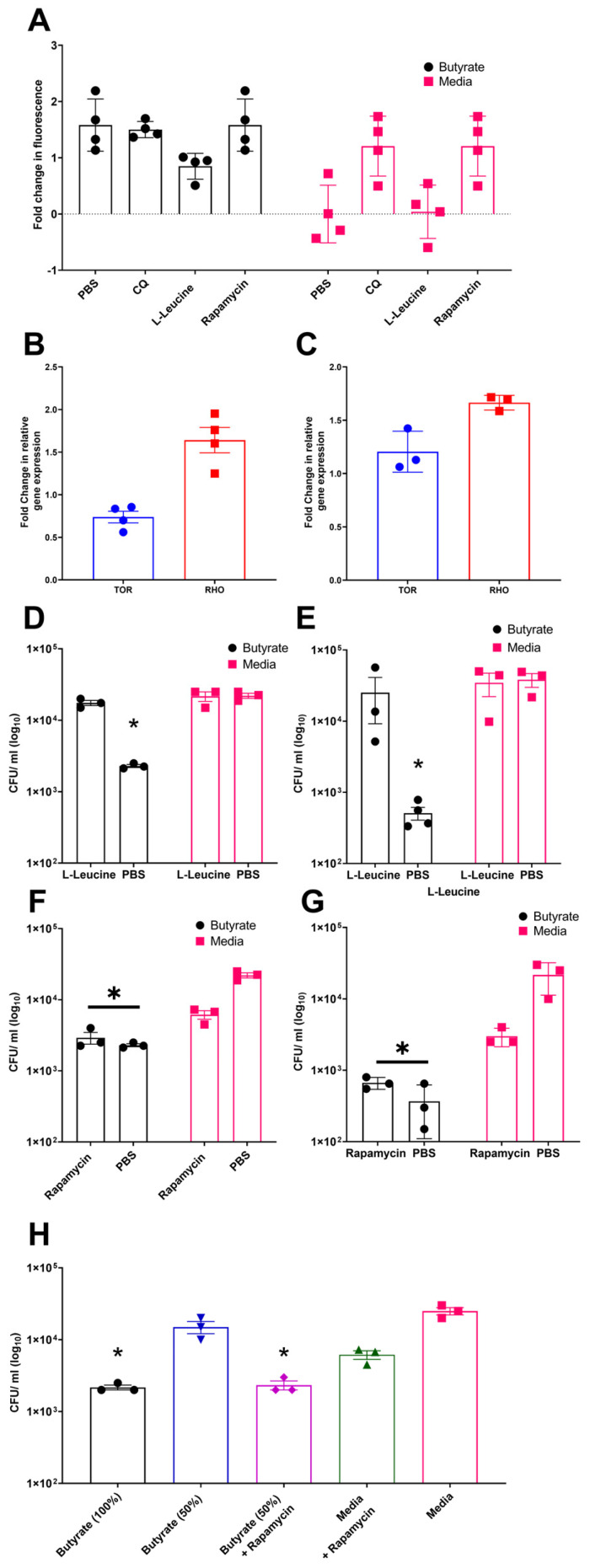
Modulation of mTOR by rapamycin and L-leucine affect LC3B and bacterial survival within butyrate-primed chicken macrophages, suggesting its involvement in the mechanism of action. (**A**) Accumulation of GFP-avLC3B in butyrate-primed and media-only HD11 exposed to 1.5 µM chloroquine (CQ), 5 µg/mL rapamycin, or 50 µg/mL L-leucine. GFP-avLC3B accumulation quantified by measurement of GFP fluorescence at excitation 488 nm and emission at 510 nm (*n* = 4). (**B**,**C**) Fold change in mTOR-associated genes TOR and RHO in (**B**) primed HD11 cells (*n* = 4) and (**C**) primed BMDMs relative to PBS-only controls (*n* = 3). (**D**,**E**) Effect of mTOR activator L-leucine (50 µg/mL) on APEC O78 ST-23 survival in butyrate-primed and media-only (**D**) HD11 and (**E**) BMDMs (*n* = 3). (**F**,**G**) Impact of mTOR inhibitor rapamycin (5 µg/mL) on APEC O78 ST-23 intracellular survival in butyrate-primed and media-only (**F**) HD11 cells and (**G**) BMDMs (*n* = 3). (**H**) Intracellular survival of APEC O78 ST-23 in HD11 cells primed with 1 mM butyrate (100%), 50% diluted butyrate (0.5 mM), and media alone, with and without the addition of 5 ug/mL rapamycin (*n* = 3). Experiments independently performed 3–4 (*n*) times. BMDMs generated from femurs of three individual birds and pooled. Data presented as mean ± SEM. Significance was determined for experiments involving grouped comparisons of three or more groups using the Kruskal–Wallis statistical test and Dunn’s test of multiple comparison. For experiments with two groups, significance was determined by Welch’s *t*-test. * = *p* ≤ 0.05.

Previous studies have demonstrated that butyrate priming of murine macrophages enhances LC3-associated autophagosome formation. This effect has been attributed to a reduction in glycolysis, which leads to inhibition of the mechanistic target of rapamycin (mTOR) pathway [29]. To investigate the role of mTOR signalling in autophagy in chicken macrophages, avLC3B-EGFP-transfected HD11 cells, either primed with butyrate or maintained in standard media, were treated with the mTOR activator L-leucine, the mTOR inhibitor rapamycin, CQ (as a positive control), or a PBS vehicle control. Consistent with mammalian studies, rapamycin and CQ increased avLC3B-associated fluorescence in control HD11 cells 2.5-fold, suggesting that mTOR inhibition induces autophagy in chicken cells. Notably, butyrate-primed HD11 cells already exhibited increased avLC3B-associated fluorescence by 2.5-fold, and subsequent exposure to rapamycin or CQ did not result in further enhancement. Conversely, treatment with the mTOR activator L-leucine reduced avLC3B-associated fluorescence in butyrate-primed cells compared to butyrate treatment alone (Figure 5A). Together, these findings support a role for mTOR pathway in the butyrate-associated accumulation of LC3, as the mTOR activator L-leucine reduces LC3 accumulation in primed cells, whereas the mTOR inhibitor triggers LC3 accumulation in media-only cells, mimicking the effect observed in butyrate-primed cells. Subsequently, we examined the influence of butyrate priming on genes involved in the mTOR pathway, specifically avian *mTOR* and *RHO* (where *RHO* upregulation is associated with mTOR pathway inhibition). We observed a decrease in *TOR* gene expression in butyrate-primed HD11 cells (Figure 5B), but unexpectedly, no significant change in BMDMs. Conversely, butyrate priming led to a moderate increase in *RHO* gene expression in both primed HD11 cells and BMDMs (Figure 5B,C) compared to the control groups.

To assess whether the effects of butyrate priming on mTOR has any role on their enhanced ability to clear bacterial killing, butyrate-primed HD11 cells or BMDMs were treated with the mTOR inhibitor rapamycin or the mTOR activator L-leucine prior to being challenged with APEC O78 ST-23. Quantification of bacterial intracellular survival using gentamicin protection assays revealed that the mTOR activator L-leucine inhibited the enhanced antimicrobial activity associated with butyrate priming in both HD11 cells (Average ± SEM: Butyrate: 2.3 × 10^3^ ± 1.1 × 10^2^ CFU/mL, Butyrate + L-Leucine: 1.75 × 10^4^ ± 1.4 × 10^3^ CFU/mL, Media: 2.2 × 10^4^ ± 1.8 × 10^3^ CFU/mL, Media + L-Leucine: 2.16 × 10^4^ ± 3.33 × 10^3^ CFU/mL, Figure 5D) and BMDMs (Average ± SEM: Butyrate: 5.1 × 10^2^ ± 1 × 10^2^ CFU/mL, Butyrate + L-Leucine: 2.5 × 10^4^ ± 1.6 × 10^4^ CFU/mL, Media: 3.8 × 10^4^ ± 8.4 × 10^3^ CFU/mL, Media + L-Leucine: 3.46 × 10^4^ ± 1.25 × 10 CFU/mL, Figure 5E). Conversely, the mTOR inhibitor rapamycin did not significantly affect bacterial survival in butyrate-primed HD11 cells (Figure 5F) or BMDMs (Figure 5G) (*p* ≥ 0.05), yet it significantly reduced intracellular bacterial survival in control HD11 cells and BMDMs (*p* < 0.05). We interpreted this to mean that the optimal butyrate concentration effectively inhibits mTOR, leading to autophagy activation, such that the subsequent addition of rapamycin provides no further enhancement. This interpretation aligns with the results presented in Figure 5A, where rapamycin failed to further increase avLC3B-associated fluorescence in butyrate-primed cells. Thus, the synergistic effect of a sub-optimal concentration of butyrate (0.5 mM) in primed HD11 cells with rapamycin were examined. The results confirmed our hypothesis and showed a synergistic reduction in intracellular bacterial survival (Average ± SEM: Butyrate 100%: 2.16 × 10^3^ ± 1.6 × 10^2^ CFU/mL, Butyrate 50%: 1.5 × 10^4^ ± 2.8 × 10^3^ CFU/mL, Butyrate 50% + Rapamycin: 3.3 × 10^2^ ± 1.8 × 10^3^ CFU/mL, Media + Rapamycin: 6.16 × 10^3^ ± 8.5 × 10^2^ CFU/mL, Media 2.5 × 10^4^ ± 2.8 × 10^3^ CFU/mL Figure 5H). The fact that the mTOR activator L-leucine reduces both LC3 accumulation and antimicrobial activity in butyrate-primed chicken macrophages, while conversely, the mTOR inhibitor rapamycin enhances LC3 accumulation and antibacterial activity (and synergistically improves clearance in sub-optimally butyrate-primed macrophages), strongly suggests that mTOR inhibition is a critical mechanism through which butyrate priming exerts its effects on chicken macrophages.

## 4. Discussion

The established ability of dietary sodium butyrate to reduce enteric pathogen colonisation in chickens presents a promising avenue for non-antimicrobial strategies for disease management in poultry, an area requiring rapid innovation [39,40]. Yet the precise mechanisms underlying this effect remain largely unexplored. While studies in mammals suggest that dietary butyrate inhibits bacterial proliferation and alters the gut environment to favour beneficial bacteria by various mechanisms—including increasing the mucosal barrier, promoting the secretion of antimicrobial peptides (AMPS), enhancing oxygen availability in the gut, modulating immune response by suppressing inflammatory response, and regulating toll-like receptors [41,42]—the dietary role of butyrate in chickens is less known. Our results suggest that sodium butyrate, at the non-cytotoxic concentrations used in this study, does not directly inhibit bacterial growth. Therefore, we examined whether sodium butyrate improves the bactericidal function of chicken macrophages and sought to understand the mechanism underlying this enhanced activity using an in vitro model. Macrophages, as key components of the innate immune system, play a critical role in the clearance of bacterial infections. Chicken macrophages have also been demonstrated to share functional similarities with mammalian macrophages, including phagocytosis, the engulfment and internalisation of bacteria, followed by their destruction within phagolysosomes. This killing is mediated by a variety of mechanisms, including the production of reactive oxygen species (ROS) through the activation of the NADPH oxidase complex, the generation of nitric oxide (NO) via inducible nitric oxide synthase (iNOS), and the release of antimicrobial peptides and enzymes [43]. Understanding how butyrate might modulate these crucial macrophage functions could provide valuable insights into its protective effects against enteric pathogens in chickens.

Our results demonstrate that sodium butyrate modulates chicken macrophage function, enhancing the in vitro bactericidal capacity of both primary and transformed chicken macrophages without impacting phagocytosis. This was observed to be a dose-dependent response, with lower butyrate concentrations resulting in the loss of enhanced bacterial clearance (Figure 5H). Yet, higher concentrations of butyrate were also observed to have a detrimental effect on cell viability as observed in previous studies [14,44]. This supports the hypothesis that sodium butyrate exhibits a dose-dependent biphasic effect on cells, supporting anti-inflammatory and regulatory functions at low concentrations (≤2 mM), whereas at higher concentrations (>5 mM) it induces cellular stress, mitochondrial dysfunction, and apoptosis [44]. This biphasic effect further complicates the application of butyrate supplementation in vivo, as the pharmacokinetics of butyrate and its final concentration in specific areas of the avian system remain poorly understood. However, oral administration of 1000–2000 mg sodium butyrate per kg feed has been shown to have a beneficial effect [45].

Intriguingly, we also observed that butyrate priming of fully differentiated bone marrow-derived macrophages failed to augment their bactericidal function. This suggests that the window of responsiveness to butyrate-induced enhancement of the bactericidal activity exerted by macrophages may be limited to macrophages undergoing differentiation. However, this does not exclude the beneficial effects of sodium butyrate via other mechanisms. This finding could have implications for the therapeutic application of sodium butyrate as an antibiotic alternative, particularly if the aim is to target already-differentiated macrophages during an active infection. To elucidate the mechanism involved in butyrate-primed macrophages, our experiments revealed that these cells exhibit an enhanced ability to produce high levels of ROS, which is essential for their improved bactericidal function. ROS directly mediates bacterial killing within macrophages by inducing various forms of damage, including DNA breaks, lipid peroxidation, and protein denaturation, leading to the disruption of bacterial cellular processes and subsequent cell death. Moreover, ROS plays a complex role in cell signalling, acting as signalling molecules and regulators of cellular processes [46]. Importantly, ROS has been shown to modulate mTOR activity in a dose- and time-dependent manner: low doses and short-term exposure stimulate mTORC1, whereas high concentrations or prolonged treatment inhibit it [47]. Analysis of mTOR-associated genes and the effects of mTOR inhibitors or activators on the function of butyrate-primed chicken macrophages suggest that mTOR is inhibited in butyrate-primed macrophages, and this plays a crucial role in the bactericidal function of the primed chicken macrophages. Similarly, reserpine, an adrenergic blocking agent, has previously been observed to result in mTOR inhibition and subsequent increases in antimicrobial responses in chicken cecal explants [48]. However, further research is required to establish the role of butyrate-induced ROS production in the modulation of mTOR activity within butyrate-primed macrophages. Furthermore, it has been shown that ROS can activate autophagy by inhibiting the PI3K-Akt-mTOR pathway or by activating AMPK to inhibit the mTOR signalling pathway [49,50,51]. Increased ROS production typically triggers autophagy as a protective mechanism to minimise intracellular damage [52]. Autophagy is crucial in macrophages for controlling bacterial infections. Through xenophagy, a selective process, lysosomes target and degrade intracellular pathogens [53]. Beyond xenophagy, autophagy extensively interacts with other host defence mechanisms, including the production and transport of antimicrobial peptides to bacterial compartments, enhancing pathogen killing [54,55]. Previous work has highlighted butyrate as an inducer of antimicrobial peptide (AMP) in chicken macrophages [11,56,57]; however, the mechanism of butyrate-induced activation and its role in the bactericidal function of butyrate-primed macrophages has yet to be determined. It is unlikely that AMP production is mutually exclusive with enhanced ROS production, as ROS can regulate cathelicidin-related antimicrobial peptide (CRAMP) expression and activity within macrophages [58]. Our results confirmed autophagy is induced in butyrate-primed chicken macrophages, and inhibition of autophagy hampered the bactericidal function of butyrate-primed chicken macrophages. This is in accordance with the results demonstrating that butyrate-induced antimicrobial activity by murine macrophages is associated with a shift in macrophage metabolism, a reduction in mTOR kinase activity, and increased autophagy [29]. While the specific pathways associated with butyrate priming of chicken macrophages are not fully understood, it could be hypothesised that the vitamin D receptor (VDR) may play an important role, as butyrate has been shown to increase intestinal VDR expression and increase autophagy and lysozyme expression [59] as well as AMP production [60]. Further research is required to examine the generation and delivery of antimicrobial peptides by butyrate-primed chicken macrophages and its influence on their antibacterial effects.

While this use of the HD11 and BMDM models provides key insights into the effect of sodium butyrate priming on the monocytes/macrophages within the avian innate immune system, it is limited, as it is unable to model the impacts of butyrate supplementation on other cell types within the innate immune system, such as heterophils and dendritic cells, as well as the adaptive immune system. Without further investigations using models of these cell types, we cannot determine if butyrate will have an equivalent beneficial effect as what has been seen within macrophages through the work performed here. It is also not possible at this stage to draw conclusions on the impact butyrate may have on the cross talk between innate and adaptive cells, an essential step necessary to facilitate the activation and expansion of the adaptive immune system following infection [61].

Currently, little is understood regarding the concentration of butyrate within the peripheral tissues outside the gut within avian species and if supplementation will increase the concentration to effective levels to induce immunomodulation. In mammals, 30 µM butyrate is typically present within the portal circulation between the gut and liver [62], the effect of supplementation and its influence on cellular responses is yet to be elucidated. In chicken, dietary supplementation of butyrate at 0.1% *wt*/*wt* results in changes in fat deposition in broilers livers, suggesting that dietary supplementation impacts areas outside the gut [63]. It is also unknown how the gut microbial community structure may impact dietary supplementation of butyrate [64]. Moreover, unlike mammals where the yolk sac is considered the major source of tissue macrophage progenitors, in birds the bone marrow has been observed to be the point of origin, from which they migrate to various tissues and become maintained as resident tissue macrophages [65]. Butyrate supplementation has previously been shown in murine models to influence cellular function within bone marrow cells, resulting in increased numbers of regulatory T cells [66] and attenuated inflammation [67,68]. This suggests that butyrate supplementation can have a pronounced effect upon the bone marrow through the gut–bone axis, potentially allowing the priming of macrophage progenitor cells during differentiation, which was observed to be necessary for the enhancement of antimicrobial activity within chicken macrophages (Figure S3) and murine models [29], prior to their migration to the peripheral tissues. Therefore, future investigations should aim to determine the effects of butyrate supplementation in vivo, with focus on determining its impact on avian immune cell responses and bone marrow macrophage progenitors. Furthermore, histone deacetylase inhibitors (HDACIs), such as butyrate [14,15], have previously been demonstrated to induce apoptosis in human cancer cell lines through the enhancement of mitochondrial ROS (mitroROS) [69], but are more well tolerated within macrophages [70]. This appears to be conserved in chickens; however, histone deacetylases (HDAC) have been shown to be essential for the viability and proliferation of the DT40 chicken B-cell line [71], suggesting further examination of the impact of butyrate on the adaptive immune system is required.

The studies presented here demonstrate that priming avian macrophages with butyrate has the capability to reduce bacterial intracellular survival by activation of ROS and inhibition of mTOR, leading to increased LC3 accumulation and the enhancement of autophagy. This effect is also not limited to APEC, with butyrate priming resulting in a reduction in the intracellular survival of multiple disparate bacterial species. Collectively, the priming of chicken macrophages with butyrate presents a possible management strategy to improve host defence against a range of bacterial pathogens. However, further investigation to the extent that this effect can be demonstrated in vivo, in addition to the potential effect on other cell types vital to the avian immune system, is required.

## Figures and Tables

**Table 1:** Bacterial isolates used within the study, consisting of APEC isolates identified as epidemiologically relevant lineages, *S.* Typhimurium SL1344, MG1655 K12 *E. coli*, MRSA NCTC 12493, and *P. aeruginosa* NCTC 12903.

Isolate Code	Phylogroup	Serogroup	Sequence Type	Source	Country of Origin
SAP 503	C	O78:H4	ST23	Layer chicken	UK
SAP 482	C	O78:H4	ST23	Layer chicken	UK
SAP 557	B2	O2:H5	ST140	Layer chicken	UK
SAP 551	B2	O2	ST140	Layer chicken	UK
SAP 487	B2	O45:H7	ST95	Layer chicken	UK
SAP 537	B2	O18:H7	ST95	Layer chicken	UK
SAP 631	G	O78	ST117	Broiler chicken	UK
SAP 641	G	O78	ST117	Broiler chicken	UK
SAP 494	G	O24:H4	ST117	Layer chicken	UK
SAP 4026	B1	O88	ST101	Turkey	UK
SAP 4027	B1	O88	ST101	Turkey	UK
MG1665			Avirulent Lab strain of *Escherichia coli* (K12)		
SL1344			*Salmonella* Typhimurium		
NCTC 12493			Methicillin resistant *Staphylococcus aureus* (MRSA)		
NCTC 12903			*Pseudomonas aeruginosa*		

**Table 2:** Primer and probe sequences for mTOR- and autophagy-associated gene expression.

Gene	GenBank Accession	Primer Sequences (5′-3′)	Reference
TOR	XM_417614	CACAACCACTGCTCGCCACAA CCATAGGATCGCCACACGGATTAG	[33]
RHO	NM_001030606.1	GTTCGGCTGGTCACGGTACATC GGTCACTTCCTTCTCTGCCTTCTG	[33]
LC3II	NM_001031461.1	GTACGAGAGCGAGAAGGACG AGACGGAAGATTGCACTCCG	[34]
ATG16L1	XM_004936938	CAAAGAACCCCTGCCTGTTG AGGGGAGACTCAGACAGACC	[35]
18S	AF173612.1	GACGACCGATTTGCACGTC GGCGAAGCCAGAGGAAA	[33]

## Data Availability

This paper does not use customised code and does not report original code. Any additional information required to re-analyse the data reported in this paper is available from the corresponding author upon reasonable request.

## Supplementary Materials

The following supporting information can be downloaded at: https://www.mdpi.com/article/10.3390/cells14211742/s1, Figure S1: Effect of differing sodium butyrate concentrations in cell culture media on HD11 viability. HD11 were seeded at 2 × 10^5^ cells/ mL in 24 well cell culture plates for 24 h in RPMI + 5% FBS + 5% CS at 41 °C with 5% CO_2_ before the addition of 1, 2, 3, 4 mM sodium butyrate, or PBS blank. Cells were incubated for 24 h before cell viability was determined by trypan blue staining. Experiments were independently performed three times, in duplicate. Error bars represent standard error of mean; Figure S2: Effect of sodium butyrate presence in cell culture media on bacterial growth kinetics. Bacterial cultures at 0.5 MacFarland standards were incubated for 18 h in RPMI + 5% FBS + 5% CS either with 1 mM butyrate (Pink) or without (Black). Optical density at 600 nm was determined every 15 min. Experiments were independently performed three times, in triplicate. Error bars represent standard error of mean; Figure S3: Effect of incubation of differentiated BMDMs with sodium butyrate on intracellular survival of APEC O78 ST-23. BMDMs were differentiated in presence of M-CSF1 for 5 days prior to exposure to 1 mM sodium butyrate for 48 h and challenge with APEC O78 ST-23 at an MOI of 10. Data presented as mean ± SEM. Experiments independently performed three times, with pooled BMDMs isolated from three birds. Significance was determined by unpaired students *t*-test.
