# Supplementary material for: Butyrate Enhances Antimicrobial Defence in Chicken Macrophages Through Reactive Oxygen Species Generation and Autophagy Activation
Source: Cells. 2025 Nov 6;14(21):1742. doi: 10.3390/cells14211742 (PMC12608662; doi:10.3390/cells14211742)
Supplement: Supplementary file 1 [file cells-14-01742-s001.zip › cells-3921020-supplementary.pdf]

Supplementary figures

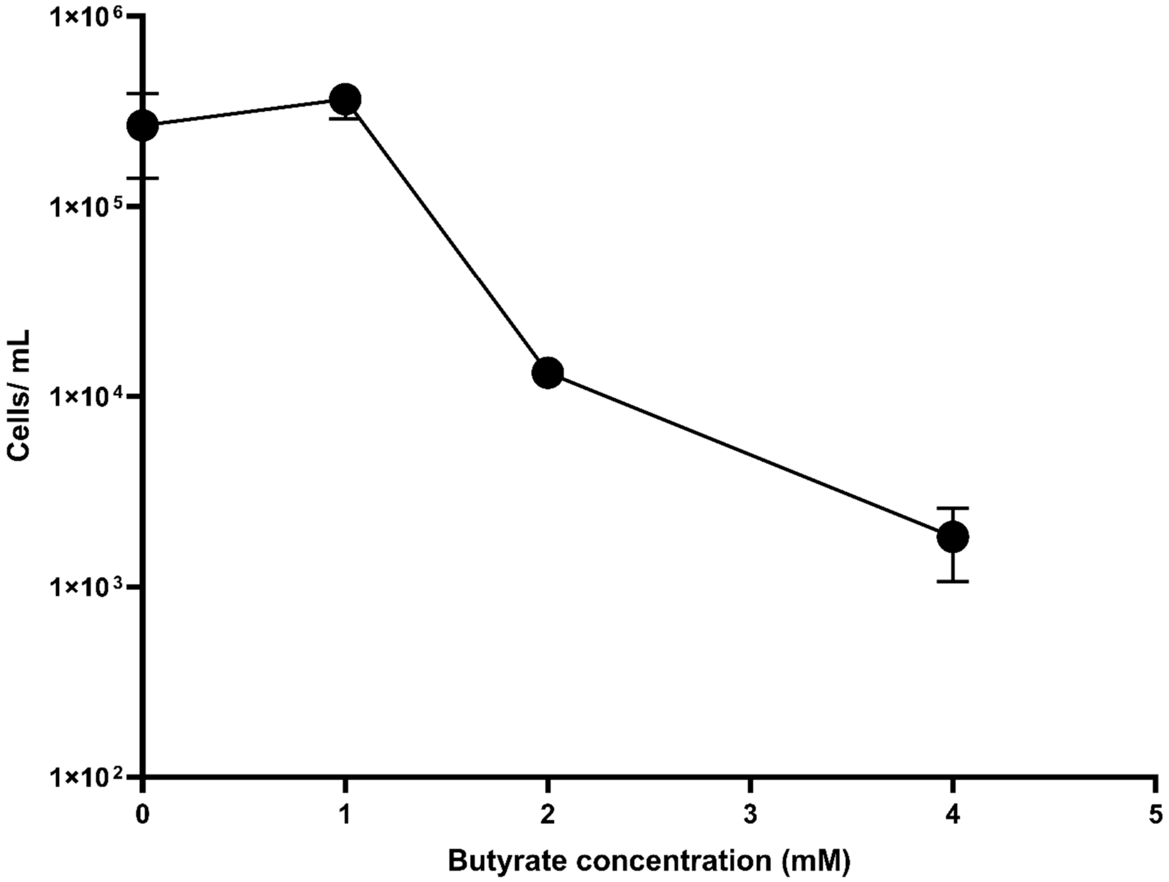

Figure S1. Effect of differing sodium butyrate concentrations in cell culture media on HD11 viability. HD11 were seeded at 2 × 10^5 cells/ mL in 24 well cell culture plates for 24 h in RPMI + 5% FBS + 5% CS at 41 °C with 5% CO_2 before the addition of 1, 2, 3, 4 mM sodium butyrate, or PBS blank. Cells were incubated for 24 h before cell viability was determined by trypan blue staining. Experiments were independently performed three times, in duplicate. Error bars represent standard error of mean.

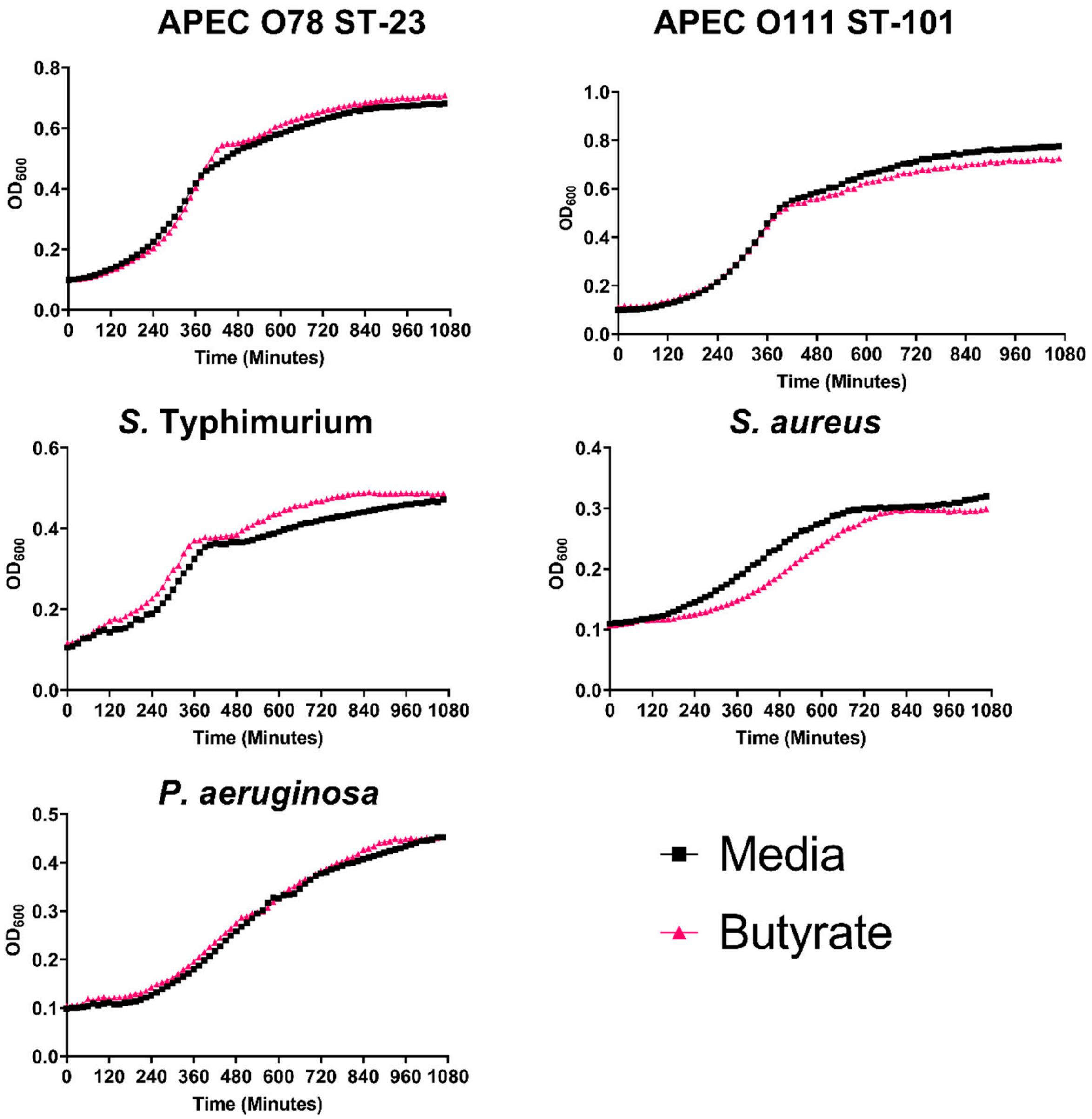

Figure S2. Effect of sodium butyrate presence in cell culture media on bacterial growth kinetics. Bacterial cultures at 0.5 MacFarland standards were incubated for 18 h in RPMI + 5% FBS + 5% CS either with 1 mM butyrate (Pink) or without (Black). Optical density at 600 nm was determined every 15 min. Experiments were independently performed three times, in triplicate. Error bars represent standard error of mean.

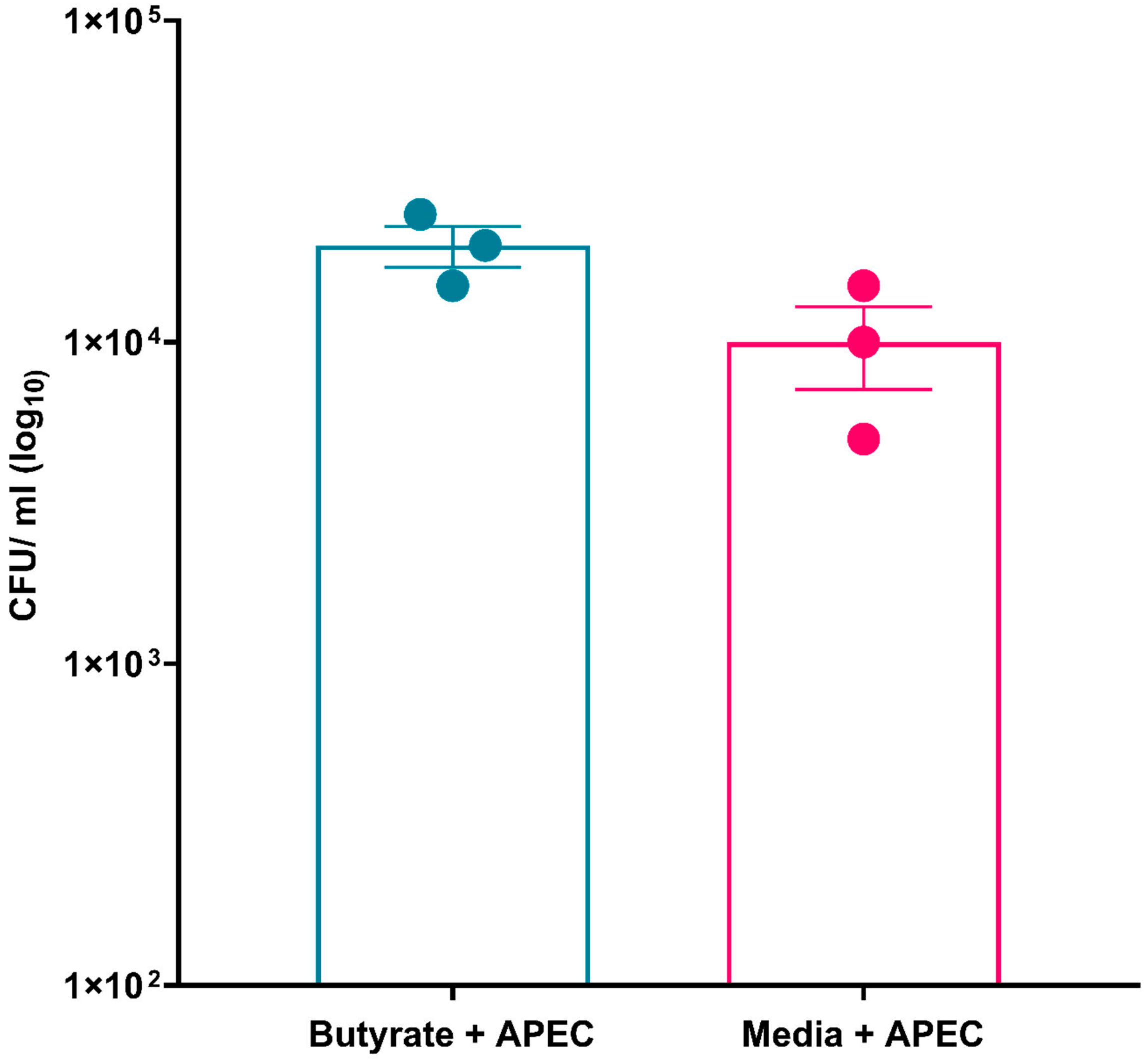

Figure S3. Effect of incubation of differentiated BMDMs with sodium butyrate on intracellular survival of APEC O78 ST-23. BMDMs were differentiated in presence of M-CSF1 for 5 days prior to exposure to 1 mM sodium butyrate for 48 h and challenge with APEC O78 ST-23 at an MOI of 10. Data presented as mean ± SEM. Experiments independently performed three times, with pooled BMDMs isolated from three birds. Significance was determined by unpaired students *t*-test.
